# Illegitimate tasks and quiet quitting: a moderated mediation model based on the strength model of self-control

**DOI:** 10.3389/fpsyg.2026.1846906

**Published:** 2026-06-23

**Authors:** Chen Liu, Zhikang Chi, Yang Sui

**Affiliations:** 1 China Women's University, Beijing, China; 2 School of Economics and Management, University of Science and Technology Beijing, Beijing, China

**Keywords:** AI usage, ego depletion, illegitimate tasks, quiet quitting, strength model of self-control

## Abstract

Quiet quitting has attracted increasing scholarly attention, yet existing research has offered limited insight into how task-level experiences are associated with employees' effort restriction. Drawing on the strength model of self-control, this study develops a moderated mediation model to examine how and when illegitimate tasks are related to quiet quitting. Using a three-wave time-lagged survey design, data were collected from 229 full-time employees in China's internet industry. The results revealed that: (1) illegitimate tasks were positively associated with ego depletion; (2) ego depletion was positively related to quiet quitting; (3) ego depletion mediated the relationship between illegitimate tasks and quiet quitting; (4) employee AI usage negatively moderated the positive relationship between illegitimate tasks and ego depletion, such that the relationship was weaker when AI usage was higher; and (5) employee AI usage moderated the indirect effect of illegitimate tasks on quiet quitting via ego depletion, such that the indirect effect was weaker at higher levels of AI usage, although the index of moderated mediation was marginal rather than conventionally significant. This study extends the antecedent research on quiet quitting by identifying a task-level driver of effort restriction, complementing existing system-level, relational, and individual-perceptual antecedents, introduces the strength model of self-control into the study of illegitimate tasks, and reveals the buffering role of employee AI usage as a coping resource, offering important implications for organizational management practice.

## Introduction

Quiet quitting has emerged as an important workplace phenomenon. Although the term has gained heightened visibility in recent years, the underlying issue is more enduring: employees may remain in their jobs while intentionally limiting their effort to the minimum required (Gray et al., 2025; Hervé and Oh, 2025). Recent scholarship has accordingly defined quiet quitting as intentionally performing only the minimum requirements of one's job while refraining from contributions beyond what is explicitly required (Gray et al., 2025; Hervé and Oh, 2025; Samnani and Robertson, 2025). This definition is theoretically important because it distinguishes quiet quitting from adjacent constructs such as disengagement, turnover intention, and social loafing. Employees who quiet quit continue to fulfill core job duties and remain formally employed, yet they consciously withhold discretionary effort and extra-role contribution (Bennett et al., 2025; Gray et al., 2025; Harris, 2025; Hervé and Oh, 2025). Emerging evidence further suggests that quiet quitting is not merely a benign individual choice; it has been linked to lower organizational citizenship behavior (OCB), higher counterproductive work behavior (CWB), reduced coworker support, and greater workplace incivility toward employees perceived as engaging in quiet quitting (Bennett et al., 2025; Gray et al., 2025; Henry et al., 2025).

Given these implications, research has increasingly examined what predicts quiet quitting, and the literature now spans several levels of analysis. At the system level, high-performance work systems have been shown to relate to quiet quitting through their effects on psychological meaningfulness and availability (Agarwal et al., 2025). At the relational level, psychological contract breach increases quiet quitting through reduced job satisfaction (Gray et al., 2025), and recurring breaches in the employer-employee relationship sustain quiet quitting over time (Georgiadou et al., 2025). At the individual-perceptual level, low perceived control over one's work life increases quiet quitting through weaker affective commitment and stronger feelings of replaceability (Hervé and Oh, 2025). Recent qualitative and bibliometric reviews further point to chronic overwork, burnout, stressful or toxic organizational cultures, and interpersonal mistreatment as candidate drivers of quiet quitting in contemporary workplaces (Harris, 2025; Jain, 2026).

These studies have made substantial progress, but they share a common feature. They characterize employees' broader work environment, their employment relationship, or their cumulative experience of work, rather than properties of the specific tasks employees are asked to do at the moment of assignment. Quiet quitting, however, is enacted task by task. Employees decide, in the flow of work, how much effort to invest in each assignment they receive. What is therefore less well-understood is whether properties of specific task assignments themselves can shape effort restriction, beyond what is explained by relational, climate-level, or aggregated experience factors. We argue that one such property is when assignments are perceived as illegitimate. Illegitimate tasks are tasks perceived as unreasonable or unnecessary because they violate norms about what can legitimately be expected from a given role (Ding and Kuvaas, 2023; Yuan et al., 2024). Although they are conceptually related to other task-level stressors such as role conflict, illegitimate tasks have been shown to explain unique variance in employee strain beyond role conflict, justice perceptions, and social stressors (Ahmed et al., 2018; Ding and Kuvaas, 2023). They confront employees with role-violating demands in the immediate flow of work, which makes them an especially proximal candidate for explaining why employees would, in the moment, reduce their discretionary effort to the minimum.

However, prior work has not adequately explained why illegitimate tasks should culminate in quiet quitting specifically. Existing research has shown that illegitimate tasks predict strain, disengagement, silence, and related withdrawal tendencies (Ding and Kuvaas, 2023; Yuan et al., 2024; Zong et al., 2022), but not why employees who remain in their jobs become inclined to cap their contribution at the minimum. This omission matters because quiet quitting is a distinctive form of behavioral recalibration: employees continue to satisfy formal role requirements while becoming increasingly unwilling to invest effort beyond them (Gray et al., 2025; Hervé and Oh, 2025). Explaining this pattern requires a theoretical lens that captures not only why illegitimate tasks are aversive, but also how they undermine employees' capacity to sustain discretionary contribution while preserving minimum compliance.

We turn to the strength model of self-control as our theoretical lens (Baumeister et al., 1998; Hagger et al., 2010; Muraven and Baumeister, 2000). The strength model proposes that self-control draws on a limited pool of psychological resources that can be consumed through sustained acts of regulation. When these resources are depleted, individuals' capacity for further self-control is reduced, a state known as ego depletion (Baumeister et al., 1998). This model is well suited to our research question for two reasons. First, illegitimate tasks confront employees with assignments that they perceive as unreasonable or beyond their role. To complete such tasks while remaining behaviorally compliant, employees have to suppress frustration, manage role-related discrepancy, and bring their behavior in line with what is asked of them. These are self-control demands that consume limited resources. Recent work on illegitimate tasks has begun to draw on the strength model to explain the resulting psychological cost (Cheng et al., 2025, 2026; Yuan et al., 2024; Zong et al., 2022). Second, the model offers a clear behavioral mechanism for quiet quitting. When self-control resources are depleted, employees find it harder to sustain effort beyond what is strictly required and tend to fall back on minimum-effort, resource-conserving behavior (Baumeister and Vohs, 2007; Muraven and Baumeister, 2000). This is the behavioral pattern that quiet quitting describes: meeting minimum role expectations while pulling back from discretionary contribution.

This reasoning points to ego depletion as the key mechanism linking illegitimate tasks to quiet quitting. Prior research suggests that illegitimate tasks require employees to expend self-control to regulate their reactions, thereby diminishing available self-regulatory resources (Baumeister et al., 1998; Yuan et al., 2024; Zong et al., 2022). In particular, illegitimate tasks can create cognitive strain, trigger negative emotional experiences, and drain employees' capacity for self-control, fostering downstream disengagement-like responses (Zong et al., 2022). Building on this logic, we argue that ego depletion is especially relevant for understanding quiet quitting because quiet quitting primarily concerns the discretionary, self-regulated portion of work contribution (Bolino et al., 2012). Depleted employees may still perform core job duties, but they are less able to sustain the initiative, attentional investment, emotional restraint, and extra-role effort required to go beyond minimum requirements (Baumeister and Vohs, 2007; Mawritz et al., 2017). Under such conditions, restricting contribution to the minimum becomes more likely. Quiet quitting, then, may be understood as a self-regulatory boundary response to diminished capacity for continued discretionary contribution.

A further question concerns when illegitimate tasks are more or less likely to produce such depletion. This issue is especially salient in contemporary workplaces, where employees increasingly rely on artificial intelligence (AI) to search for information, structure workflows, and handle routine procedural work. Prior research suggests that AI-related experiences are not uniformly beneficial: they may facilitate work-goal progress (Bankins et al., 2024; Li et al., 2024; Sun et al., 2025), but they may also heighten self-esteem threat and related strain (Ren and Chowdhury, 2025; Tang et al., 2023). Relatedly, research on task-AI fit suggests that AI can support task execution when it aligns with task requirements, but can also trigger threat when it undermines employees' sense of value or fit (Cheng et al., 2025; Lu and Currie, 2026). Our theorizing focuses on a narrower construct: employee AI usage as a task-enactment behavior, rather than organizational AI deployment or generalized AI threat perceptions. To the extent that employees use AI to process information, organize materials, draft outputs, or handle repetitive components of work, the cognitive and procedural burden associated with illegitimate assignments may be reduced. In turn, the self-regulatory effort required to absorb role discrepancy while remaining compliant may also be lower. Put differently, employee AI usage may lessen the self-regulatory cost of coping with illegitimate tasks, thereby weakening the positive relationship between illegitimate tasks and ego depletion.

**Figure 1:**
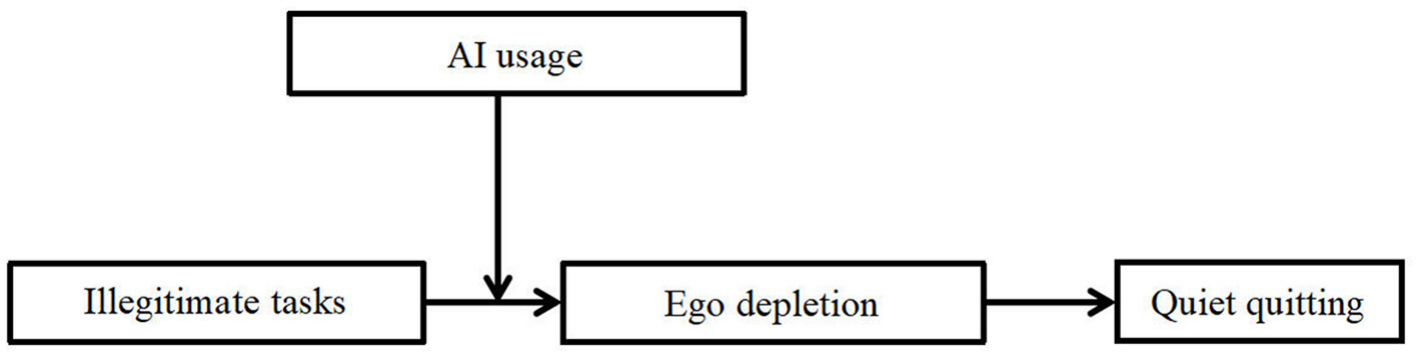
Research model.

Our aim in this study is to examine how and when illegitimate tasks are associated with quiet quitting. Specifically, we develop a moderated mediation model in which illegitimate tasks are related to quiet quitting through ego depletion, and employee AI usage attenuates the first-stage relationship between illegitimate tasks and ego depletion. Using a three-wave field study of full-time employees in China, we make three contributions. First, we advance quiet quitting research by shifting its antecedent logic from broad relational appraisals to a more proximal, task-level driver, illegitimate tasks, thereby offering a more fine-grained account of how concrete work experiences shape employees' effort recalibration. Second, we introduce the strength model of self-control as a unifying process explanation that clarifies not only why illegitimate tasks are aversive but also how they culminate in quiet quitting via ego depletion. The proposed research model is presented in (Figure 1). Third, we theorize employee AI usage not merely as a technological backdrop but as a coping-relevant task-support condition that can lower the self-regulatory cost of dealing with adverse task assignments. Together, these contributions offer a more precise account of when illegitimate tasks are associated with quiet quitting and when that tendency may be mitigated.

The remainder of this article is organized as follows. We first present our theoretical framework, grounded in the strength model of self-control, and develop the hypotheses. We then describe the three-wave field study, the sample, and the measures. Next, we report the results of the measurement model, the structural analyses, and the test of moderated mediation. Finally, we discuss the theoretical and practical implications of the findings and note limitations and directions for future research.

## Theory and hypotheses

### The strength model of self-control and ego depletion

The strength model of self-control proposes that self-control depends on a limited and depletable pool of psychological resources (Baumeister et al., 1998; Hagger et al., 2010; Muraven and Baumeister, 2000). Across a wide range of regulatory acts, including emotion regulation, cognitive inhibition, behavioral restraint, and impulse control, individuals draw on this common resource. As the resource is consumed, the capacity to perform subsequent acts of self-control declines, a state referred to as ego depletion (Baumeister et al., 1998). Ego depletion has been shown to weaken executive functioning, reduce motivation, and increase the likelihood of effort minimization in subsequent tasks (Baumeister and Vohs, 2007; Hagger et al., 2010).

We adopt the strength model for three reasons. First, illegitimate tasks place direct demands on employees' self-control. To complete tasks that they perceive as unreasonable or unnecessary, employees have to suppress dissatisfaction, manage role-related frustration, and bring their behavior in line with what is asked of them. This sustained regulatory effort consumes self-control resources and increases the likelihood of ego depletion, a relationship supported by prior research (Cheng et al., 2026; Yuan et al., 2024; Zhou et al., 2018; Zong et al., 2022). Second, ego depletion provides a clear behavioral mechanism for quiet quitting. When self-control resources are depleted, employees become less able to sustain the discretionary effort that goes beyond minimum role requirements (Baumeister and Vohs, 2007; Muraven and Baumeister, 2000), and resource-conservation tendencies make minimum-effort responses more likely. Third, the strength model provides a natural lens for theorizing employee AI usage as a boundary condition. To the extent that employees can use AI to handle parts of their work, the regulatory effort required to cope with illegitimate task demands is reduced, which in turn slows the depletion process.

In sum, the strength model offers a single, internally consistent framework for our hypotheses: illegitimate tasks deplete employees' self-control resources and produce ego depletion; ego depletion increases the likelihood of quiet quitting as a low-effort, resource-conserving response; and employee AI usage attenuates this process by lowering the resource cost of dealing with illegitimate task demands.

### Illegitimate tasks and ego depletion

Building on the antecedent landscape outlined above, we now develop the mechanism through which illegitimate tasks, as a task-level role violation, deplete employees' self-control resources. According to the strength model of self-control, individuals' capacities for self-control, emotion regulation, and behavioral inhibition all depend on a finite pool of psychological resources. As these resources are continuously expended, individuals are increasingly likely to experience a depleted state characterized by diminished self-control capacity and psychological fatigue, namely ego depletion (Baumeister et al., 1998; Baumeister and Vohs, 2007). When carrying out work tasks that violate role norms or extend beyond the boundaries of reasonable job responsibilities, employees must regulate their reactions and maintain task engagement, drawing on their limited self-control resources in the process. Such sustained regulatory effort accelerates resource exhaustion, thereby increasing the likelihood of ego depletion (Yuan et al., 2024).

Illegitimate tasks are defined as work demands that violate employees' role expectations and are perceived as unreasonable or unnecessary, encompassing both unnecessary and unreasonable tasks (Semmer et al., 2010). Encountering such tasks requires employees to suppress frustration, manage role-related discrepancy, and bring their behavior in line with what is asked of them despite perceiving the demands as inappropriate. These regulatory efforts draw on the same limited pool of self-control resources that the strength model describes, and sustained exposure to them increases the likelihood of ego depletion (Yuan et al., 2024; Zong et al., 2022).

Empirical evidence has directly supported the effect of illegitimate tasks on ego depletion. Using a daily diary design, Yuan et al. (2024) found that the more illegitimate tasks employees perceived on a given day, the more likely they were to experience immediate ego depletion. Similarly, Zong et al. (2022) and Dong and Zhang (2022) demonstrated that illegitimate tasks, by occupying cognitive and emotional resources, serve as a critical stress antecedent triggering ego depletion. Moreover, illegitimate tasks signal disrespect and unfairness, which adds to employees' psychological burden and intensifies the self-regulatory demands of the situation (Semmer et al., 2015; Schulte-Braucks et al., 2019). Taken together, the evidence suggests that illegitimate tasks draw consistently on employees' self-control resources, thereby increasing their susceptibility to ego depletion.

**Hypothesis 1:** Illegitimate tasks are positively related to ego depletion.

### Ego depletion and quiet quitting

The strength model of self-control posits that self-control and psychological regulation depend on finite resources. When these resources are continuously expended without timely recovery, individuals enter a state of ego depletion, characterized by reduced impulse control, weakened behavioral monitoring, lower motivation, and insufficient volitional effort (Baumeister et al., 1998; Hagger et al., 2010). Employees in a depleted state struggle to sustain high levels of self-regulation and are more likely to adopt effort-minimizing, low-effort strategies that minimize further resource expenditure to conserve remaining resources (Muraven and Baumeister, 2000).

Quiet quitting refers to employees' deliberate restriction of work effort to the minimum required by their job roles, avoidance of extra tasks, refusal to exert discretionary effort, and strict adherence to work boundaries. At its core, quiet quitting involves deliberately scaling back effort to avoid further resource expenditure (Hervé and Oh, 2025; Kanwal et al., 2026). From a self-regulation perspective, ego depletion increases the likelihood of quiet quitting by lowering employees' capacity to sustain proactive and effortful work behaviors.

First, ego depletion undermines proactive work motivation. Depleted employees experience reductions in intrinsic and achievement motivation, making it difficult to sustain proactive engagement and high performance. Instead, they tend to limit their effort to the minimum required to maintain their job, rather than pursuing excellence or taking on extra responsibilities (Baumeister et al., 2007). In a resource-depleted state, the regulatory cost of proactive behaviors such as initiative, collaboration, or continuous improvement rises sharply, prompting a shift toward quiet quitting (Hervé and Oh, 2025).

Second, ego depletion impairs behavioral self-control and role adherence. Reduced self-regulatory capacity makes it harder for employees to overcome fatigue, resistance, and withdrawal tendencies, undermining their ability to maintain high standards and prompting psychological and behavioral exit strategies. As Kanwal et al. (2026) note, quiet quitting encompasses both passive disengagement and deliberate effort reduction, and the regulatory failures induced by ego depletion constitute a core psychological antecedent of these behaviors.

Finally, employees who are depleted tend to protect remaining self-control resources by drawing in their work boundaries. They are less likely to attend non-mandatory meetings, respond to emails outside working hours, or take on tasks beyond the minimum required. These patterns map directly onto the behavioral profile that quiet quitting describes (Hervé and Oh, 2025; Kanwal et al., 2026).

Empirical evidence supports the foregoing logic. Ego depletion has been shown to significantly reduce work engagement, organizational citizenship behaviors, and proactive behaviors, while increasing withdrawal tendencies and the propensity to perform only minimal required duties (Zhou et al., 2018; Yuan et al., 2024). Research on quiet quitting further confirms that insufficient psychological resources and diminished self-control constitute key psychological mechanisms underlying employees' decision to limit effort (Hervé and Oh, 2025; Kanwal et al., 2026).

Taken together, the higher the level of ego depletion, the more limited employees' self-regulatory resources become, reducing their capacity to maintain high work engagement and increasing the likelihood of adopting quiet quitting as a resource-conservation strategy.

**Hypothesis 2:** Ego depletion is positively related to quiet quitting.

Building on the logic of H1 and H2, we propose that ego depletion mediates the relationship between illegitimate tasks and quiet quitting. This mediation claim warrants further elaboration because illegitimate tasks could, in principle, affect quiet quitting through other pathways, such as negative emotions or perceived injustice (Ding and Kuvaas, 2023). What makes ego depletion a particularly fitting mediator is the match between its downstream behavioral consequences and the specific form of effort restriction that defines quiet quitting.

Consider the nature of quiet quitting. Unlike counterproductive work behavior, which involves active rule-breaking, or turnover, which involves leaving the organization, quiet quitting involves meeting minimum role expectations while withholding the discretionary, self-controlled portion of work contribution (Bennett et al., 2025; Gray et al., 2025; Hervé and Oh, 2025). Sustaining discretionary effort requires ongoing self-regulation: employees must maintain initiative, exercise attentional control, manage fatigue, and invest in tasks beyond what is strictly necessary (Bolino et al., 2012). These are precisely the capacities that ego depletion undermines. When self-control resources are depleted, employees do not necessarily stop working altogether, but they become less able to sustain the additional effort that goes beyond minimum compliance (Baumeister and Vohs, 2007; Muraven and Baumeister, 2000). The resulting behavioral pattern, continued formal role performance coupled with withdrawal of discretionary contribution, maps directly onto what quiet quitting describes.

Alternative mediators such as negative emotions or injustice perceptions would not predict this specific pattern as cleanly. Negative emotions may lead to counterproductive behavior, interpersonal conflict, or turnover intention rather than to calibrated effort restriction (Zhou et al., 2018). Injustice perceptions may motivate retaliation or voice rather than quiet compliance at the minimum level (Ahmed et al., 2018). Ego depletion, by contrast, specifically reduces the capacity for sustained discretionary effort while leaving basic role performance relatively intact, which makes it the most theoretically precise mechanism for explaining quiet quitting as an outcome of illegitimate tasks.

**Hypothesis 3:** Ego depletion mediates the relationship between illegitimate tasks and quiet quitting.

### The moderating effect of employee AI usage

The strength model implies that whether illegitimate tasks deplete employees' self-control resources depends on how much regulatory effort employees actually have to spend on them. We propose that employee AI usage shapes this cost, and that the relationship between illegitimate tasks and ego depletion is therefore weaker for employees who use AI more in their work. Before developing this argument, however, we acknowledge that AI at work is not uniformly beneficial. A growing body of research shows that employees can also experience AI as a source of strain. Working closely with intelligent machines can deplete self-control resources through sustained mental engagement with the technology itself (Tang et al., 2023). AI adoption can be appraised as a hindrance rather than a challenge, depending on how it is introduced and what it signals about the employee's role (Cheng et al., 2023). Digital transformation can also threaten self-esteem and produce mixed outcomes for job attitudes (Lu and Currie, 2026; Ren and Chowdhury, 2025). Whether AI helps or hinders therefore depends on the specific construct under examination and on the work demands employees face (Bankins et al., 2024).

Our argument focuses on a narrower construct: employee AI usage as a task-enactment behavior. Following Yuan et al. (2026), we define this as the extent to which employees themselves draw on AI tools to process information, draft outputs, organize materials, or handle routine procedural work in their daily tasks. This construct differs from organizational AI adoption or implementation, which reflects top-down decisions about technology deployment (Cheng et al., 2023), and from generalized perceptions of AI as a threat to one's job, status, or sense of self (Ren and Chowdhury, 2025; Tang et al., 2023). The distinction matters for our purposes because our outcome of interest is the self-control resource cost of coping with illegitimate task demands, which is shaped most directly by what employees do with AI in the moment, not by the broader organizational or psychological framing of the technology.

We argue that employee AI usage attenuates the relationship between illegitimate tasks and ego depletion through two complementary mechanisms. The first is task substitution. Illegitimate tasks often include components that are repetitive, information-intensive, or procedural in nature, such as compiling materials, formatting documents, drafting routine communications, or searching for background information. Employees who use AI tools can offload these components to the technology, which means that the volume of effortful self-regulation required to complete the assignment is reduced. This logic is consistent with research portraying AI as a collaborator that takes on parts of the task and leaves the employee to focus on the rest (Jia et al., 2024; Sun et al., 2025; Wilson and Daugherty, 2018), and with evidence that employee-driven AI engagement supports rather than replaces human effort (Li et al., 2024).

The second mechanism is resource conservation. Even when AI does not directly substitute for parts of an illegitimate task, employees who use AI in their daily work tend to complete their legitimate, role-appropriate tasks more efficiently. This frees self-control resources that would otherwise be spent on routine work and leaves employees with more capacity to absorb the regulatory demands of unreasonable assignments when they arise. This logic follows from Yuan et al.'s (2026) account of AI usage as a dual self-regulation mechanism, in which AI both takes over part of the task and helps employees conserve resources for other demands, and from broader work portraying AI as a job resource (Bankins et al., 2024; Li et al., 2024).

Together, these two mechanisms suggest that employee AI usage lowers the self-control resource cost of dealing with illegitimate task demands. When employees rely heavily on AI in their work, part of that cost is absorbed by the technology rather than by the employee, so the relationship between illegitimate tasks and ego depletion should be weaker. When employees use AI less, they have to absorb the full cost on their own, and the relationship should be stronger.

**Hypothesis 4:** Employee AI usage moderates the positive relationship between illegitimate tasks and ego depletion, such that the relationship is weaker when AI usage is higher.

Integrating the mediating logic of H3 with the moderating logic of H4, the indirect positive effect of illegitimate tasks on quiet quitting via ego depletion is expected to be negatively moderated by AI usage. Specifically, when AI usage is high, the indirect effect of illegitimate tasks on quiet quitting through ego depletion is weaker; conversely, when AI usage is low, this indirect effect is stronger.

**Hypothesis 5:** AI usage moderates the indirect effect of illegitimate tasks on quiet quitting via ego depletion, such that the indirect effect is weaker when AI usage is higher and stronger when AI usage is lower. The full hypothesized research model is presented in (Figure 1).

## Materials and methods

### Data collection

We recruited a sample of full-time knowledge workers employed at internet-sector firms in China. Prior to data collection, all participants were fully informed about the purpose of the study and provided their voluntary consent to participate. Participants were told that they could withdraw from the study at any time without any negative consequences. No personally identifiable information was collected, and responses were matched across waves using anonymous participant ID codes. This study involved only standard survey measures of routine workplace experiences and did not include any sensitive populations, deceptive procedures, or interventions with potential for harm. We adopted a time-lagged, multi-wave survey design to mitigate common method bias and to establish the temporal ordering implied by our theoretical model (Podsakoff et al., 2003). Data were collected at three time points, each separated by approximately 2 weeks. At Time 1 (T1), participants reported on their experience of illegitimate tasks and their use of AI tools at work. At Time 2 (T2), 2 weeks later, the same participants reported on their level of ego depletion. At Time 3 (T3), another 2 weeks later, participants completed measures of quiet quitting.

We matched responses across waves using unique anonymous participant IDs. We began with 268 employees who agreed to participate at T1. After excluding individuals who withdrew from the study or who provided incomplete responses at any wave, the final analytic sample comprised *N* = 229 employees, yielding a retention rate of 85.4% across the three waves. The final sample had the following demographic profile: 67.2% were women (*n* = 154) and 32.8% were men (*n* = 75). The mean age was 32.07 years (*SD* = 6.62). With respect to educational attainment, 22.3% held a diploma or associate degree, 72.9% held a bachelor's degree, and 4.8% held a graduate degree. Participants had an average organizational tenure of 6.39 years (*SD* = 5.95).

### Measurement

Established scales were used to measure all focal variables. Because the study was conducted in China, the English items were translated into Chinese and back-translated following Brislin's (1980) procedure. A bilingual researcher with prior collaborative experience translated the items into Chinese, and a second bilingual researcher, who was unfamiliar with the original items, independently back-translated the Chinese version into English. Minor discrepancies were discussed and resolved with the translators to ensure semantic equivalence. Unless otherwise noted, items were rated on Likert-type scales following the response formats of the original scales. Illegitimate tasks, AI usage, and ego depletion were rated on 7-point scales ranging from 1 = strongly disagree to 7 = strongly agree, whereas quiet quitting was rated on a 5-point scale ranging from 1 = strongly disagree to 5 = strongly agree. The full list of items is provided in Appendix A.

Illegitimate tasks were measured at T1 using the scale developed by Semmer et al. (2010). The scale contains eight items, four of which measure unnecessary tasks, such as “Do you have work tasks to take care of, which keep you wondering if they have to be done at all?”, and four of which measure unreasonable tasks, such as “Do you have work tasks to take care of, which you believe should be done by someone else?” The reliability coefficient for the scores for this sample was 0.954.

AI usage was measured at T1 using three items from Tang et al. (2022). A sample item is “I used artificial intelligence to carry out most of my job functions.” The reliability coefficient for the scores for this sample was 0.897.

Ego depletion was measured at T2 using five items from the scale developed by Twenge et al. (2004). A sample item is “My mental energy is running low.” The reliability coefficient for the scores for this sample was 0.959.

Quiet quitting was measured at T3 using the five-item scale developed by Hervé and Oh (2025). A sample item is “At work, I consciously choose not to work beyond my primary job responsibilities.” The reliability coefficient for the scores for this sample was 0.908.

## Data analysis

Data analysis was conducted in three stages using complementary statistical software. First, descriptive statistics and correlations were computed in SPSS 26.0. Second, confirmatory factor analysis (CFA) was performed using Mplus 8.3. Third, we tested the hypothesized measurement and structural models within a unified Structural Equation Modeling (SEM) framework using lavaan 0.6-21 in R 4.2.2 (Rosseel, 2012), with maximum likelihood (ML) estimation and 5,000 bias-corrected bootstrap resamples for all coefficient and indirect-effect inference (Preacher and Hayes, 2008).

### Descriptive statistics and correlational analysis

Table 1 presents the means, standard deviations, and zero-order correlations among the study variables. Illegitimate tasks were positively correlated with AI usage (*r* = 0.313, *p* < 0.01), ego depletion (*r* = 0.369, *p* < 0.01), and quiet quitting (*r* = 0.204, *p* < 0.01). Ego depletion was positively correlated with quiet quitting (*r* = 0.298, *p* < 0.01), consistent with the proposed mediation pathway. Notably, AI usage was not significantly correlated with quiet quitting (*r* = 0.038, *p* > 0.05), which is consistent with the prediction that AI usage operates as a boundary condition on the illegitimate tasks–ego depletion relationship rather than as a direct antecedent of quiet quitting.

**Table 1:** Means, standard deviations, and intercorrelations among study variables.

Variables	Mean	*SD*	1	2	3
1. Illegitimate tasks	3.169	1.312			
2. AI usage	3.640	1.436	0.313^**^		
3. Ego depletion	3.169	1.376	0.369^**^	0.172^**^	
4. Quiet quitting	2.357	0.939	0.204^**^	0.038	0.298^**^

N = 229. ^**^p < 0.01 (two-tailed).

The pattern of bivariate correlations is broadly consistent with our hypothesized model and provides initial, unconditional support for the mediation and moderation pathways we test in the subsequent analyses.

### Measurement model

Because all measures were obtained from a single source (self-report), we took several steps to evaluate the potential for common method bias (CMB). First, by design, we separated the measurement of predictors (T1), the mediator (T2), and the outcome (T3) across three temporally distinct waves, each separated by 2 weeks. This procedural remedy substantially reduces, though does not entirely eliminate, the risk of CMB inflating observed relationships (Podsakoff et al., 2003).

Second, we conducted Harman's single-factor test as a *post hoc* diagnostic. We entered all items from the four focal scales into an exploratory factor analysis and examined the unrotated factor solution. The first unrotated factor accounted for 38.53% of the variance in the item pool, which is below the conventional threshold of 50%, providing initial evidence that CMB is not a primary concern in this dataset (Podsakoff et al., 2003).

Third, we re-examined the four-factor measurement model linking illegitimate tasks, AI usage, ego depletion, and quiet quitting. The measurement model showed acceptable fit on CFI, TLI, and SRMR (see Table 2), *χ*^2^_(183)_ = 539.481, *p* < 0.001, CFI = 0.921, TLI = 0.909, RMSEA = 0.092, SRMR = 0.041, although RMSEA exceeded the conventional 0.08 threshold. All standardized factor loadings were large and significant, ranging from 0.76 to 0.93 (all *ps* < 0.001). Composite reliability was excellent for all four constructs, and AVE values exceeded the 0.50 threshold (Fornell and Larcker, 1981), supporting convergent validity (see Table 3). Heterotrait–Monotrait (HTMT) ratios for all six construct pairs were below the conservative 0.85 threshold (range = 0.040-0.385; Henseler et al., 2015), supporting discriminant validity (see Table 4). We further compared the hypothesized four-factor model against more parsimonious alternatives. The four-factor model fit significantly better than all three alternative models (Δχ^2^ tests all *ps* < 0.001), confirming that the four constructs are empirically distinguishable. Taken together, these analyses support the distinctiveness of our constructs and suggest that CMB is unlikely to account for the key findings.

**Table 2:** Results of confirmatory factor analysis (CFA).

Model	*N*	χ^2^	*df*	Δχ^2^	*Δdf*	χ^2^/*df*	Δχ^2^/*df*	CFI	TLI	RMSEA	SRMR
Four-factor model	229	539.481	183	—	—	2.948	—	0.921	0.909	0.092	0.041
Three-factor model	229	1690.164	186	1150.683	3	9.087	6.139	0.666	0.623	0.188	0.160
Two-factor model	229	2435.259	188	1895.778	5	12.954	10.006	0.502	0.443	0.228	0.212
Single-factor model	229	2769.254	189	2229.773	6	14.652	11.704	0.428	0.364	0.244	0.202

**Table 3:** Reliability and validity estimates.

Construct	α	CR (ω)	AVE
Illegitimate tasks	0.954	0.951	0.721
AI usage	0.897	0.898	0.746
Ego depletion	0.959	0.959	0.826
Quiet quitting	0.908	0.909	0.670

α = Cronbach's alpha. CR, composite reliability; AVE, average variance extracted. All loadings are significant at *p* < 0.001.

**Table 4:** Discriminant validity (HTMT ratios).

Construct	1	2	3	4
1. Illegitimate tasks				
2. AI usage	0.331			
3. Ego depletion	0.385	0.184		
4. Quiet quitting	0.212	0.040	0.314	

HTMT, Heterotrait-Monotrait ratio of correlations. All values are below the conservative 0.85 threshold, supporting discriminant validity among the four constructs.

### Main effect and mediation analysis

We tested H1–H3 using a latent-variable mediation model in which illegitimate tasks predicted ego depletion and quiet quitting, and ego depletion predicted quiet quitting. Bias-corrected 95% confidence intervals (CIs) were obtained from 5,000 bootstrap resamples. The model showed adequate fit on CFI, TLI, and SRMR, though RMSEA was above the conventional cutoff, *χ*^2^_(132)_ = 465.05, *p* < 0.001, CFI = 0.918, TLI = 0.905, RMSEA = 0.105, SRMR = 0.041. Consistent with H1, illegitimate tasks were positively and significantly associated with ego depletion 2 weeks later, *b* = 0.400, *SE* = 0.082, *z* = 4.87, *p* < 0.001, 95% CI [0.242, 0.562], β = 0.390. Employees who reported higher levels of unnecessary or unreasonable tasks reported substantially greater self-regulatory resource depletion. Consistent with H2, ego depletion positively predicted quiet quitting after controlling for the direct effect of illegitimate tasks, *b* = 0.176, *SE* = 0.054, *z* = 3.28, *p* = 0.001, 95% CI [0.075, 0.286], β = 0.280. The direct effect of illegitimate tasks on quiet quitting, once ego depletion was modeled, was reduced and no longer significant, *b* = 0.076, *SE* = 0.053, z = 1.42, *p* = 0.156, 95% CI [−0.029, 0.176], β = 0.118, consistent with ego depletion mediating the relationship between illegitimate tasks and quiet quitting. Consistent with H3, the bootstrapped indirect effect of illegitimate tasks on quiet quitting via ego depletion was positive and significant, *ab* = 0.070, *SE* = 0.029, 95% CI [0.023, 0.137], with the CI excluding zero. The total effect was also significant, *c* = 0.146, *SE* = 0.054, 95% CI [0.042, 0.254]. These results support a mediation model in which ego depletion serves as the psychological mechanism linking illegitimate tasks to quiet quitting (see Table 5).

**Table 5:** Results of the latent mediation model (*N* = 229).

Path	*b*	*SE*	*z*	*p*	95% CI	β
Direct paths						
Illegitimate tasks → ego depletion (a)	0.400^***^	0.082	4.87	< 0.001	[0.242, 0.562]	0.39
Ego depletion → quiet quitting (b)	0.176^**^	0.054	3.28	0.001	[0.075, 0.286]	0.28
Illegitimate tasks → quiet quitting (c')	0.076	0.053	1.42	0.156	[−0.029, 0.176]	0.118
Indirect and total effects						
Indirect effect (a × b)	0.070^*^	0.029	2.42	0.016	[0.023, 0.137]	0.109
Total effect (c)	0.146^**^	0.054	2.69	0.007	[0.042, 0.254]	0.227

β = standardized regression coefficient. 95% CIs are bias-corrected based on 5,000 bootstrap resamples. c′ denotes the direct effect of illegitimate tasks on quiet quitting controlling for ego depletion; c denotes the total effect. ^*^p < 0.05, ^**^p < 0.01, ^***^p < 0.001.

### Moderation and moderated mediation analysis

To test the moderating role of AI usage (H4), we estimated a latent moderation model using orthogonalized matched product indicators (Little et al., 2006; Marsh et al., 2004). Specifically, we computed three matched product indicators by multiplying the mean of the centered illegitimate-tasks indicators with each centered AI usage indicator and residualized each product on its constituent terms. The model showed adequate fit on CFI, TLI, and SRMR, though RMSEA marginally exceeded the conventional 0.08 cutoff, $\chi_{\left(244\right)}^{2}$ = 612.34, *p* < 0.001, CFI = 0.929, TLI = 0.920, RMSEA = 0.081, SRMR = 0.041. The main effect of illegitimate tasks on ego depletion remained robust, *b* = 0.380, *SE* = 0.084, *z* = 4.53, *p* < 0.001, 95% CI [0.209, 0.540], β = 0.370. The main effect of AI usage was non-significant, b = 0.060, SE = 0.079, *p* = 0.449. As expected, the latent illegitimate tasks × AI usage interaction was negative, *b* = −0.102, *SE* = 0.052, *z* = −1.97, *p* = 0.048, 95% CI [−0.204, 0.001], β = −0.177. This pattern provides modest support for H4 and indicates that AI usage attenuates the depleting effect of illegitimate tasks on ego depletion. Although the upper bound of the bias-corrected bootstrap 95% CI is at the boundary of zero, the z-test result (*p* = 0.048) and the directional consistency of the effect across the robustness check (*b* = −0.092, *p* = 0.088 with controls) provide convergent support for H4 (see Table 6). The negative interaction indicates that AI usage attenuates the depleting effect of illegitimate tasks on ego depletion.

**Table 6:** Results of the latent moderated mediation model (*N* = 229).

Predictor	Ego depletion (mediator)			Quiet quitting (DV)		
	*B(β)*	*SE*	95% CI	*Bβ*	*SE*	95% CI
Illegitimate tasks	0.380^***^(0.370)	0.084	[0.209, 0.540]	0.076(0.117)	0.054	[−0.029, 0.176]
AI usage	0.060(0.063)	0.079	[−0.092, 0.216]	—	—	—
Illegitimate tasks × AI usage	−0.102^*^(−0.177)	0.052	[−0.204, 0.001]	—	—	—
Ego depletion	—	—	—	0.176^**^(0.280)	0.054	[0.075, 0.286]
*R* ^2^	0.187			0.117		

B(β) = unstandardized coefficient with standardized coefficient in parentheses. 95% CIs are biascorrected based on 5,000 bootstrap resamples. Latent interaction estimated via orthogonalized matched product indicators. ^*^p < 0.05, ^**^p < 0.01, ^***^p < 0.001.

**Figure 2:**
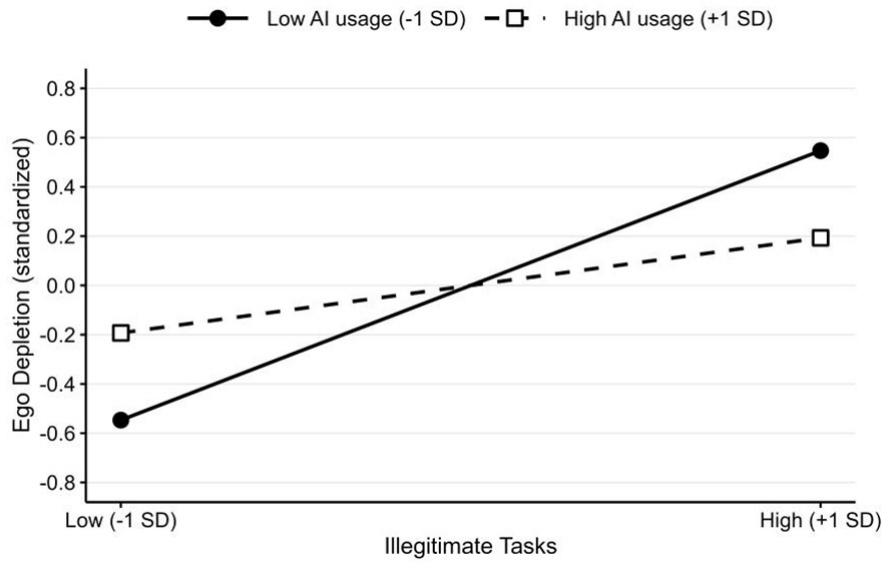
The moderating effect of AI usage.

To probe the form of the interaction, we computed simple slopes at one standard deviation above and below the latent mean of AI usage (Aiken and West, 1991). At low levels of AI usage (−1 SD), illegitimate tasks were strongly and positively associated with ego depletion, *b* = 0.482, *SE* = 0.080, *z* = 6.05, *p* < 0.001, 95% CI [0.321, 0.635]. At high levels of AI usage (+1 SD), the slope, while still positive, was substantially attenuated, *b* = 0.278, *SE* = 0.114, *z* = 2.43, *p* = 0.015, 95% CI [0.049, 0.494]. The pattern, displayed in Figure 2, shows that AI tools buffer employees against the self-regulatory cost of illegitimate task demands.

We then tested the full moderated mediation model (H5). The conditional indirect effects followed the hypothesized pattern (see Table 7). At low levels of AI usage (−1 SD), the conditional indirect effect of illegitimate tasks on quiet quitting via ego depletion was positive and significant, ab = 0.085, *SE* = 0.032, *p* = 0.008, 95% CI [0.032, 0.154]. At high levels of AI usage (+1 SD), the conditional indirect effect was smaller, ab = 0.049, *SE* = 0.029, 95% CI [0.005, 0.117], representing an approximately 42% reduction from the low-AI-usage condition. The index of moderated mediation was negative and approached conventional significance, index = −0.036, *SE* = 0.020, *z* = −1.83, *p* = 0.067, 95% CI [−0.077, 0.000] (Hayes, 2015). Because the formal index did not meet the 0.05 threshold, we interpret H5 as partially supported: the conditional indirect relationships followed the hypothesized pattern, with the indirect relationship being weaker at high than at low AI usage, but the index of moderated mediation itself was marginally significant rather than conventionally significant.

**Table 7:** Conditional indirect effects and index of moderated mediation (*N* = 229).

Pathway: illegitimate tasks → ego depletion → quiet quitting	Indirect effect	*SE*	95% CI
Low AI usage (−1 SD)	0.085	0.032	[0.032, 0.154]
High AI usage (+1 SD)	0.049	0.029	[0.005, 0.117]
Index of moderated mediation	−0.036	0.020	[−0.077, 0.000]

Conditional indirect effects and the index of moderated mediation are based on 5,000 bias-corrected bootstrap resamples within a latent-variable SEM framework.

### Robustness checks

To address potential concerns about whether our pattern of findings is contingent upon the inclusion of demographic controls (Bernerth and Aguinis, 2016), we re-estimated the full latent moderated mediation model with gender, age, education, job type, and tenure entered simultaneously as covariates predicting both ego depletion and quiet quitting. The pattern of focal effects was substantively unchanged: the effect of illegitimate tasks on ego depletion remained significant (β = 0.374, *p* < 0.001), and the effect of ego depletion on quiet quitting remained significant (β = 0.265, *p* = 0.002), and the illegitimate tasks × AI usage interaction remained directionally identical (β = −0.160, *p* = 0.088). The conditional indirect effect at low AI usage remained significant (ab = 0.079, 95% CI [0.027, 0.146]), while the conditional indirect effect at high AI usage was smaller and approached but did not clearly exceed conventional thresholds (ab = 0.048, 95% CI [0.005, 0.118]). The model fit was acceptable, χ^2^_(354)_ = 738.51, CFI = 0.926, TLI = 0.917, RMSEA = 0.069 (90% CI [0.062, 0.076]), SRMR = 0.042. This robustness check confirms that our findings are not artifacts of the controls specification (Becker, 2005; Spector and Brannick, 2011). Among the controls, only age was a significant negative predictor of ego depletion (β = −0.240, *p* = 0.003), consistent with the literature on age-related self-regulatory development; this pattern does not affect any of our focal hypotheses.

## Discussion and conclusion

### General discussion and conclusion

This study extends emerging research on quiet quitting by showing how a concrete, everyday work experience is associated with employees' restriction of effort to the minimum required. Building on the strength model of self-control, we found that illegitimate tasks are related to quiet quitting through ego depletion and that employee AI usage weakens the positive relationship between illegitimate tasks and ego depletion. We further found that the conditional indirect relationship was weaker at higher levels of AI usage, although the formal index of moderated mediation was only marginal rather than conventionally significant. In doing so, our study responds to recent calls to move beyond broad accounts of quiet quitting centered on psychological contract dynamics or perceived control and to identify more proximal, work-based drivers of this phenomenon (Gray et al., 2025; Hervé and Oh, 2025). Our findings suggest that employees do not quiet quit only because they develop diffuse negative evaluations of their organization. They may also do so because the work they are asked to perform repeatedly violates role expectations and requires them to regulate frustration, resentment, and compliance in the immediate flow of work.

These findings connect two literatures that have thus far developed largely in parallel. Quiet quitting research has increasingly clarified the construct as a deliberate restriction of effort rather than a synonym for disengagement, low OCB, or turnover intention (Gray et al., 2025; Hervé and Oh, 2025). At the same time, illegitimate-task research has shown that role-inappropriate, unnecessary, or unreasonable assignments predict strain, negative emotions, disengagement, silence, and related withdrawal tendencies (Ding and Kuvaas, 2023; Yuan et al., 2024; Zong et al., 2022). By linking these streams, our study suggests that one important downstream consequence of illegitimate tasks is not necessarily immediate exit or total withdrawal, but a more calibrated retreat from discretionary contribution. Employees remain in place and continue to perform essential duties, yet they become less willing or less able to invest the self-regulatory effort required to go beyond them. This pattern is consistent with recent work portraying quiet quitting as a nuanced boundary-setting response rather than uniform passivity or simple detachment (Bennett et al., 2025; Georgiadou et al., 2025; Gray et al., 2025).

Our findings also speak to the contemporary relevance of AI in shaping employee responses to problematic work demands. The AI-at-work literature has documented that AI can support task execution, provide cognitive job resources, and enable proactive role-shaping, but can also heighten identity threat, insecurity, or dehumanization under some conditions (Bankins et al., 2024; Li et al., 2024; Ren and Chowdhury, 2025; Tang et al., 2023). By focusing specifically on employee AI usage rather than organizational AI deployment, our study clarifies one way in which AI may mitigate rather than intensify employee strain. One plausible interpretation of our findings is that, when employees use AI to handle information-intensive or repetitive aspects of work, they may preserve more of the self-control resources needed to remain behaviorally engaged. In this sense, AI may shape employee outcomes not only by altering performance or threat perceptions, but also by changing the self-control resource cost of task enactment. We caution, however, that this buffering pattern should not be assumed to apply to all forms of AI use. The same literature also shows that AI engagement can deplete self-control through sustained mental coordination with the technology (Tang et al., 2023), can be appraised as a hindrance rather than a resource (Cheng et al., 2023), and can introduce identity- or job-related threats (Ren and Chowdhury, 2025). Conditions under which employee AI usage shifts from buffering to amplifying strain are an important topic for future research.

### Theoretical contributions

Our study makes three primary theoretical contributions.

First, we contribute to the quiet quitting literature by identifying a task-level driver of effort restriction. Prior research has established that quiet quitting is shaped by system-level conditions such as high-performance work systems (Agarwal et al., 2025), by relational evaluations such as psychological contract breach (Georgiadou et al., 2025; Gray et al., 2025), and by individual-perceptual appraisals such as low perceived control over one's work life (Hervé and Oh, 2025), as well as by broader experiences of overwork, burnout, and stressful organizational cultures captured in recent reviews (Harris, 2025; Jain, 2026). What this literature has not closely examined is whether properties of specific task assignments can themselves prompt employees to recalibrate their effort. Our study addresses that gap by identifying illegitimate tasks as a proximal, task-level antecedent of quiet quitting. Unlike system, relational, or aggregated experience factors, illegitimate tasks operate in the immediate flow of work. They confront employees with assignments that appear unreasonable, unnecessary, or inconsistent with their role, thereby bringing the question of effort boundaries into sharp relief in the moment of task assignment (Ahmed et al., 2018; Ding and Kuvaas, 2023; Yuan et al., 2024). This matters because quiet quitting is itself enacted task by task: employees continue to meet formal job requirements while intentionally withholding discretionary contribution on specific assignments (Bennett et al., 2025; Gray et al., 2025; Hervé and Oh, 2025). By showing that task-level role violations help explain such behavior, our study advances quiet quitting research from a focus on broad employer-employee evaluations to a more fine-grained account centered on specific task experiences.

Second, we contribute to both the illegitimate-task literature and the quiet quitting literature by adopting the strength model of self-control as a unifying process explanation. Prior work on illegitimate tasks has drawn on stress-as-offense-to-self, justice, affective, and resource-based perspectives (Ding and Kuvaas, 2023). These perspectives have generated important insights, but they do not directly explain why role-violating work demands should culminate in quiet quitting specifically. The strength model fills this gap because it specifies a clear behavioral mechanism: sustained self-control demands deplete a limited resource, and once depleted, employees are less able to maintain the discretionary effort that goes beyond minimum role requirements (Baumeister et al., 1998; Hagger et al., 2010; Muraven and Baumeister, 2000). Recent work has begun to apply this logic to illegitimate tasks (Cheng et al., 2026; Yuan et al., 2024), and we extend it by linking the depletion process to quiet quitting as a downstream outcome. In doing so, we reframe illegitimate tasks not simply as aversive demands, but as demands that draw down employees' self-control resources, with predictable consequences for the kind of effort employees are still able to sustain.

This finding clarifies why ego depletion is especially relevant for understanding quiet quitting. Prior research has already shown that illegitimate tasks can drain self-control resources and foster downstream disengagement-like responses (Yuan et al., 2024; Zong et al., 2022). Our findings extend this logic by showing that quiet quitting can emerge through precisely this process. Once employees' self-regulatory resources are depleted, the discretionary, self-controlled portion of work contribution becomes harder to sustain even if minimum compliance remains intact. Quiet quitting is therefore not simply an attitudinal reflection of dissatisfaction; it is also a behavioral manifestation of reduced capacity for continued discretionary contribution. This contribution helps position quiet quitting more precisely at the intersection of role expectations, self-regulatory strain, and effort boundary setting.

Third, we contribute to the literature on AI at work by theorizing employee AI usage as a coping-relevant task-support condition rather than merely a technological backdrop. Current AI research increasingly emphasizes heterogeneity in employee reactions. AI can improve efficiency, provide cognitive job resources, and enable proactive role-shaping behaviors such as AI crafting, but it can also trigger threat, insecurity, and dehumanization under some conditions (Bankins et al., 2024; Li et al., 2024; Ren and Chowdhury, 2025; Tang et al., 2023). Our study builds on this insight by showing that whether AI alleviates or exacerbates strain depends in part on what, exactly, is being theorized. We focus on employee AI usage as a task-enactment behavior, that is, how employees themselves use AI to complete work, rather than on top-down AI implementation or generalized AI threat perceptions. This distinction enables a more precise theoretical account.

Specifically, our findings suggest that employee AI usage can reduce the self-regulatory cost associated with coping with illegitimate tasks. This contribution moves beyond portraying AI as uniformly empowering or threatening. Instead, it identifies a narrower, behaviorally grounded function of AI in the work process. To the extent that employees use AI to handle information-intensive or repetitive components of work, they may preserve self-control resources that would otherwise be consumed in coping with those tasks. In this way, AI usage may shape employee outcomes not only through performance enhancement or threat generation, but also by altering how much self-regulatory effort is required to perform undesired work.

Taken together, these contributions advance understanding of quiet quitting, illegitimate tasks, and AI at work by placing them within a common explanatory framework. Our core theoretical claim is that role-violating task demands can become self-regulatory burdens, those burdens can deplete employees' capacity for continued discretionary effort, and employee AI usage can shape how costly that process becomes.

### Practical implications

Our findings also offer several practical implications for organizations and managers.

First, organizations should treat illegitimate tasks as more than minor annoyances or temporary inconveniences. Because such tasks can contribute to quiet quitting by draining self-regulatory resources, organizations should systematically identify where role-violating demands enter employees' workflows. This may require clarifying job boundaries, auditing recurring extra tasks, reducing unnecessary procedural burdens, and ensuring that work is assigned to the most appropriate role holder. Prior illegitimate-task research likewise suggests that organizations can reduce the harmful effects of such tasks through effective job design, clearer task boundaries, and more transparent communication about why particular tasks are necessary (Ahmed et al., 2018; Cheng et al., 2026; Ding and Kuvaas, 2023). Even when such tasks appear small in isolation, repeated exposure can accumulate into meaningful self-regulatory strain and ultimately reduce employees' willingness to contribute beyond the minimum.

Second, managers should recognize that quiet quitting may be preceded by regulatory fatigue rather than by immediate disloyalty or detachment. In practice, this means that efforts to address quiet quitting solely by exhorting employees to be more committed or proactive may be misguided. A more effective response is to ask whether employees are repeatedly being required to absorb role discrepancy and maintain compliance under conditions that deplete self-control. This implication is consistent with research showing that quiet quitting is related to reduced extra-role contribution, lower job satisfaction, higher exhaustion, and weaker work relationships, but is not interchangeable with those constructs (Bennett et al., 2025; Georgiadou et al., 2025; Gray et al., 2025). Managers should therefore monitor signs such as frustration, emotional flatness, reduced helping, or reluctance to take on discretionary tasks, and then consider whether role-inappropriate assignments are contributing to those patterns.

Third, our finding that employee AI usage buffers the strain from illegitimate tasks should be interpreted with caution. The implication is not that organizations should deploy AI tools to help employees tolerate poor job design. Our results show that AI usage attenuates the relationship between illegitimate tasks and ego depletion, but does not eliminate it: illegitimate tasks remained a significant predictor of ego depletion even at high levels of AI usage. The more appropriate reading is from the employee perspective. Employees who already use AI in their daily work appear better positioned to preserve self-control resources when they face unreasonable task demands, suggesting that integrating AI into one's own workflow may serve as a personal coping resource (Li et al., 2024; Yuan et al., 2026). However, the primary practical response to quiet quitting should remain the reduction of illegitimate tasks at the source through better job design, fairer task allocation, and clearer role boundaries. Organizations that rely on AI to compensate for poor task management risk normalizing role violations and expanding the volume of unreasonable work, which would ultimately undermine the buffering effect we observed.

In sum, reducing quiet quitting requires more than boosting general morale. It requires attention to the concrete work experiences that shape employees' willingness and capacity to contribute beyond the minimum. By minimizing illegitimate tasks, recognizing the self-regulatory burden they impose, and understanding that employees' own AI usage may serve as a personal coping resource, organizations can better protect employees' capacity for discretionary contribution and reduce the likelihood that quiet quitting becomes a stable response.

### Limitations and future research directions

Our findings should be interpreted in light of several limitations that also open useful directions for future research. First, although our three-wave field design strengthens temporal separation among key variables, it does not fully capture the short-term, within-person dynamics through which illegitimate tasks may accumulate into ego depletion and, eventually, quiet quitting. Quiet quitting is likely not a single episode, but a gradual recalibration of effort that unfolds as employees repeatedly encounter role-violating demands and regulate their reactions over time. Future research could therefore complement our design with experience-sampling, diary, or hybrid experimental-field approaches to examine whether repeated exposure to illegitimate tasks produces escalating self-regulatory costs and whether such processes are more acute on days when employees face unusually high role discrepancy (Yuan et al., 2024; Zong et al., 2022).

Second, our study focused on full-time employees in China. This context is valuable given the rapid diffusion of AI tools and the salience of role obligations in Chinese workplaces, but the meaning of illegitimate tasks, the interpretation of effort boundaries, and the acceptance of AI-assisted work may vary across institutional and cultural settings. Future research could examine whether our theorized process generalizes across contexts that differ in role norms, managerial expectations, and technological maturity. Comparative designs may be especially helpful in clarifying whether the relationship between illegitimate tasks and quiet quitting is stronger in settings where extra-role effort is more strongly normalized or where AI use is more deeply embedded in everyday work (Georgiadou et al., 2025; Hervé and Oh, 2025; Tang et al., 2023).

Third, our theorizing treated employee AI usage as a task-enactment behavior that lowers the self-regulatory cost of coping with illegitimate tasks. That focus was theoretically useful, but it necessarily compresses meaningful heterogeneity in AI use. Future research could unpack whether different forms of AI usage, such as routine automation support, generative drafting, decision assistance, or collaboration with more autonomous tools, operate in similar ways. It may also be fruitful to examine when AI use ceases to be buffering and begins to amplify role discrepancy, for example when it increases task volume, blurs accountability, or heightens perceived threat. Doing so would extend the dual self-regulation logic articulated by Yuan et al. (2026) and connect more directly to recent work on the conditions under which AI is experienced as a resource vs. a threat (Cheng et al., 2023; Lu and Currie, 2026; Ren and Chowdhury, 2025). Relatedly, future research could differentiate more carefully between passive and deliberate forms of quiet quitting (Kanwal et al., 2026) and examine whether ego depletion is more predictive of the former than the latter. Such work would further refine understanding of when quiet quitting reflects depleted regulatory capacity vs. a more strategic boundary-setting choice.

Fourth, our time-lagged design, while temporally separating the measurement of predictors, mediator, and outcome, does not constitute a true longitudinal panel design in which the same focal variables are measured repeatedly. Therefore, we cannot draw definitive causal conclusions, and the findings should be interpreted as evidence of temporally ordered associations rather than definitive causal effects. Future research should employ full panel designs or experimental manipulations to examine causality more rigorously.

## Data Availability

The raw data supporting the conclusions of this article will be made available by the authors, without undue reservation.

## Appendix A. Measurement items

**Illegitimate tasks (T1;** **Semmer et al., 2010**)

*Unnecessary tasks Stem: Do you have work tasks to take care of, which keep you wondering if…*

1. they have to be done at all?
2. they make sense at all?
3. they just exist because some people simply demand it this way?
4. they would not exist, or could be done with less effort, if things were organized differently?

*Unreasonable tasks Stem: Do you have work tasks to take care of, which you believe…*

5. should be done by someone else?
6. should be done in a different context, e.g., by another professional group?
7. are going too far, and should not be expected from you?
8. put you in an awkward position?

**AI usage (T1; Tang et al., 2022)**

1. I used artificial intelligence to carry out most of my job functions.
2. I spent most of the time working with artificial intelligence.
3. I worked with artificial intelligence in making major work decisions.

**Ego depletion (T2; Twenge et al., 2004)**

1. I feel drained.
2. My mind feels unfocused right now.
3. Right now, it would take a lot of effort for me to concentrate on something.
4. My mental energy is running low.
5. I feel like my willpower is gone.

**Quiet Quitting (T3; Hervé and Oh, 2025)**

1. At work, I consciously choose not to work beyond my primary job responsibilities.
2. No matter how urgent a task is, it is only right for me to work on it during official work hours.
3. At work, I choose not to work extra hours to enhance the quality of my work beyond the minimum expected of me.
4. I choose not to check work-related emails after work hours if I am not required to do so.
5. At work, I choose not to attend non-mandatory meetings.
